# Nutritional and Functional Properties of Fermented Mixed Grains by Solid-State Fermentation with *Bacillus amyloliquefaciens* 245

**DOI:** 10.3390/foods9111693

**Published:** 2020-11-19

**Authors:** Su Jin Heo, Ah-Jin Kim, Min-Ju Park, Kimoon Kang, Do Yu Soung

**Affiliations:** 1 Food Research Institute, CJ Cheil Jedang, 42, Gwanggyo-ro, Yeongtong-gu, Suwon-si, Gyeonggi-do 16495, Korea; sujin.heo@cj.net (S.J.H.); ahjin.kim@cj.net (A.-J.K.); kimoon.kang@cj.net (K.K.); 2 BIO Research Institute, CJ Cheil Jedang, 42, Gwanggyo-ro, Yeongtong-gu, Suwon-si, Gyeonggi-do 16495, Korea; minju.park@cj.net

**Keywords:** solid-state fermentation, *Bacillus amyloliquefaciens*, grain, metabolites, enzymatic activity

## Abstract

Fermented foods have several advantages, including increased nutritional value, improved bioavailability, and functional health properties. We examined that these outcomes were also observed in fermented mixed grains (FMG) containing wheat germ, wheat bran, oats, brown rice, barley, quinoa, and lentils following solid-state fermentation (SSF) by *Bacillus amyloliquefaciens* 245. The metabolic profile during fermentation was screened using capillary electrophoresis time-of-flight mass spectrometry (CE-TOF-MS). The amino acids were quantitatively measured for the validation of the changes in metabolites. The activity of enzymes (e.g., amylase, protease, and fibrinolysis) and antioxidant capacity was also assessed to elucidate the functionality of FMG. The essential amino acid contents gradually increased as fermentation progressed. As the metabolites involved in the urea cycle and polyamine pathway were changed by fermentation, arginine was used as a substance to produce citrulline, ornithine, and agmatine. FMG showed dramatic increases in enzyme activity. FMG incubated for 36 h also displayed higher total phenolic contents and free radical scavenging ability than MG. The data suggest that FMG produced by *Bacillus amyloliquefaciens* 245 possess improved nutritional and functional quality, leading to their potential use as dietary supplements.

## 1. Introduction

Wheat (*Triticum aesticum* L.) is one of the most consumed grains in the world. The usage of wheat was 760.2 million tonnes from the production of 771.5 million tonnes (USDA) in 2019/20 [1]. Throughout the processing chain of wheat flour, a large quantity of byproducts including the germ and bran is generated. Although wheat bran is rich in dietary fiber, phenolic acids, and proteins, its bioavailability is limited by the insoluble cell layer structure and high content of anti-nutritional factors such as phytate [2]. Similarly, wheat germ has high nutritional value, being a source of vitamin E, B vitamins, protein, dietary fiber, minerals, unsaturated fatty acids, and functional phytochemicals. However, its consumption is also restricted by anti-nutritional substances and existing lipase activity [3].

To overcome these issues, microbial fermentation has been noted for the successful use of wheat and other grain byproducts in the application of functional foods. For example, fermentation has extensively decomposed the cell wall structure of wheat bran, leading to an increase in soluble arabinoxylans and digestible protein [4]. Rizzello et al. demonstrated that lower lipase activity in fermented wheat germ than raw wheat germ effectively reduced the technological obstacles in baking [5]. The fermented quinoa flour also significantly degraded phytate while improving the bioavailability of zinc and iron [6]. Through fermentation, barley and oat bran were used to obtain xylitol and folate which is employed in sugar-free products and maternity dietary supplements, respectively [7]. Additionally, the heat-stabilized defatted rice bran during fermentation released more phenolic compounds such as gentistic, caffeic, syringic, p-coumaric, ferulic, sinapic, and benzoic acids than the control [8].

A choice of microbial bioprocesses and microorganisms suitable for fermentation is an important factor that strengthens the nutritional and functional properties of grain byproducts. Solid-state fermentation (SSF) requires only a small amount of water, close to that found in the natural environment of the microorganisms. This allows SSF to exert a positive economic and environmental impact when used in place of conventional submerged fermentation [9]. Some examples of foods fermented using SSF are cheonggukjang, natto, tempeh, fermented enzyme products, and kimchi. Among the microorganisms that may be used for SSF, *Bacillus amyloliquefaciens* rather than *Lactobacillus* and *Saccharomycess*, decreased the amount of anti-nutritional factors and increased the level of antioxidant activity in fermented soybean meal [10]. *Bacillus amyloliquefaciens* also produced higher levels of amino acids than *Aspergillus oryzae*, in soybeans, wheat, and rice [11]. Thus, SSF with *Bacillus amyloliquefaciens* might be a good candidate for the fermentation of grains.

We have characterized *Bacillus amyloliquefaciens* 245 isolated from Nuruk, a traditional Korean fermentation starter that produces rice-based wine. Its Genbank accession number is KR535604 [12]. We demonstrated that among 1008 *Bacillus* strains from either Nuruk or soybeans, *Bacillus amyloliquefaciens* 245 was one of the best in terms of amylase, protease, and fibrinolytic activity [13]. In particular, the largest amount of amino acids was observed when the mixed grain (MG)—including wheat germ, wheat bran, oats, brown rice, barley, quinoa, and lentils—was used as the substrate for SSF with *Bacillus amyloliquefaciens* 245 compared to when individual grains were used on a same-total basis [13]. We performed scientific metabolic profiling and investigated the nutritional and functional value of MG fermented by *Bacillus amyloliquefaciens* 245 during the different stages of SSF.

## 2. Materials and Methods

### 2.1. Materials

The MG consisted of wheat germ, wheat bran, oats, brown rice, barley, quinoa, and lentils at a weight ratio of 40:30:10:5:5:5:5. We used *Bacillus amyloliquefaciens* 245 isolated from Nuruk, a traditional Korean fermentation starter that produces rice-based wine.

### 2.2. Solid-State Fermentation (SSF)

The MG was steamed at 100 °C for 30 min in an autoclave. After inoculation with a 10% of *Bacillus amyloliquefaciens* 245 in the sterilized mixed grain (*w/w*), the fermented MG (FMG) was incubated for 0, 8, 12, and 36 h at a constant temperature (37 °C) in a chamber under 95% humidity. The concentration of *Bacillus amyloliquefaciens* 245 was 5.0 × 10^9^–1.5 × 10^10^ CFU/mL. The FMG was dried at 60 °C for 12 h, crushed using a cyclone mill, and then used for analysis.

### 2.3. Metabolic Profiling Using Capillary Electrophoresis Time of Flight Mass Spectrometry (CE-TOF-MS) Analysis

Samples were mixed with 600 μL of methanol containing internal standards (50 μM) using a homogenizer (1500 rpm for 120 s, three times). After adding chloroform (600 μL) and distilled water (240 μL), the homogenates were thoroughly mixed and centrifuged (2300× *g*, 4 °C, 5 min). The aqueous layer was filtered through a 5-kDa cut-off filter (ULTRAFREE-MC-PLHCC, Human Metabolome Technologies, Yamagata, Japan) to remove macromolecules, and the filtrate was centrifuged and resuspended in 50 μL of distilled water immediately before the measurement. As previously described [14,15], the anionic and cationic metabolites in the samples were analyzed using an Agilent CE-TOF-MS system (Agilent Technologies, Santa Clara, CA, USA) equipped with fused silica capillaries in a 50 μm (i.d.) × 80 cm CE-MS column. Conditions were as follows: buffer solutions were used; MS capillary voltages, 3500 V (anion mode) and 4000 V (cation mode); MS scan range, *m/z* 50–1000; and the Human Metabolome Technologies (HMT) sheath liquid was used. The peaks detected in the CE-TOF-MS analysis were extracted using automatic integration software (Master Hands ver. 2.17.1.11, developed at Keio University, Keio, Japan), and the peak area was converted to a relative peak area. Putative metabolites were assigned using HMT’s standard and known–unknown peak libraries on the basis of the *m/z* and migration time. The tolerance was ±0.5 min in MT and ±10 ppm in *m/z*. Hierarchical cluster analysis (HCA) was performed using statistical analysis software.

### 2.4. Free Amino Acid Analysis

Samples (1 g) were extracted with 20 mL of water by shaking for 1 h. The extract was centrifuged at 12,000× *g* for 5 min. The supernatant was then filtered through a 0.22 μm membrane filter and injected into an HPLC (high-performance liquid chromatography) column. Each amino acid was analyzed using two mobile phases as follows [16]. Mobile Phase A was a solution of 10 mM Na_2_HPO_4_, 10 mM Na_2_B_4_O_7_, and 5 mM NaN_3_ in 1 L of distilled water at pH 8.2. Mobile Phase B was a mixture of acetonitrile, methanol, and water at a volumetric ratio of 45:45:10. Pre-column derivatization was performed in the autosampler and the programmable injector units. Then, 2.5 μL of 0.1 M borate buffer (Agilent Technologies, Palo Alto, CA, USA) were mixed with 1 μL of sample by drawing air five times. After 0.2 min, 0.5 μL of ortho-phthalaldehyde, 0.4 μL of 9-fluorenylmethyl chloroformate, and 32 μL of injection diluent reagents were added, mixed eight times by drawing air, and injected after 0.1 min of waiting. Separation was carried out using an Agilent ZORBAX Eclipse Plus C18, 4.6 × 150 mm, 3.5 μm particle size i.d. HPLC column with detection at 338 nm. The gradient program started with 2% Phase B at a flow rate of 1.8 mL/min and changed linearly between 0.5 and 18 min to 51.3% Phase B. Next, 100% Phase B was applied from 18.1 to 21.5 min, and then, 98% of Phase A was applied until the end of the program at 25 min.

### 2.5. Quantitative Estimation of Targeted Arginine Metabolites Using HPLC

Arginine metabolites were analyzed using the method described by Caro et al. [17], with modifications. Samples (1 g) were extracted with 20 mL of distilled water by shaking for 1 h. The extract was centrifuged at 12,000× *g* for 5 min. The supernatant was remixed with 20 mL of 0.01 N HCl, filtered through a 0.22 μm membrane filter, and injected into an HPLC column. The HPLC analysis was performed using the autosampler (the Agilent 1200 series, Agilent Technologies, Palo Alto, CA, USA) equipped with a ZORBAX Eclipse Plus C18, 4.6 × 150 mm, 3.5 μm particle size i.d. HPLC column and a UV (DAD) detector at 40 °C with detection at 338 nm. The mobile phase consisted of a mixture of Mobile Phases A (50 mM sodium phosphate buffer, pH 6.8) and B (6:2:2 mixture of methanol, acetonitrile, and water). Separation was obtained at a flow rate of 1 mL/min with a gradient program that allowed for 0 min at 20% Phase B followed by 10 min of increasing Phase B to 80%.

### 2.6. Determination of Enzymatic Activity

#### 2.6.1. Sample Extraction

Each sample (5 g) was extracted with 95 mL of distilled water by shaking at 180 rpm for 5 min. The mixture was centrifuged at 1628× *g* for 5 min, and then, the supernatant was filtered through Whatman No. 2 filter paper.

#### 2.6.2. Amylase Activity Assay

Amylase activity was determined as described by Pfueller and Elliott [18]. One mL of the extracted sample was mixed with 13 mL of 0.1 N sodium phosphate buffer (pH 7.0), 1 mL of 0.1% calcium chloride, and 5 mL of 1% soluble starch solution. The mixture was incubated at 37 °C for 30 min and added to a color reagent solution. Absorbance was measured at 660 nm. One unit (U) of amylase activity is defined as the amount of amylase required to hydrolyze 10 mg of starch per 30 min.

#### 2.6.3. Protease Activity Assay

Protease activity was assayed using the method described by Kum et al. [19], with modifications. One mL of the extracted sample was mixed with 1 mL of 0.6% casein and incubated at 37 °C for 10 min. The reaction was stopped by adding 2 mL of 0.4 M trichloroacetic acid and incubating at 37 °C for 25 min. The mixture was centrifuged at 13,000× *g* for 5 min. An amount of 1 mL of each supernatant was mixed with 5 mL of 0.4 M sodium carbonate, and then 1 mL of thrice-diluted 2 N Folin reagent was added. The mixture was incubated at 37 °C for 20 min. The absorbance was measured at 660 nm. One unit of protease activity is defined as the amount of protease required to release 1 μg of tyrosine per min.

#### 2.6.4. Fibrinolytic Activity Assay

Fibrinolytic activity was measured according to the method described by Chang et al. [20], with modifications. A 100 μL volume of extract mixed with 300 μL of 0.1 M Tris-HCl (pH 7.8) containing 10 mM CaCl_2_ was incubated in a 30 °C water bath for 5 min. After adding 300 μL of 1.2% fibrin solution (pH 7.8) prepared in 0.5 N NaOH, the mixture was incubated at 30 °C for another 10 min. The reaction was stopped by adding 600 μL of 0.11 M trichloroacetic acid containing 0.22 M sodium acetate and 0.33 M acetic acid. After centrifugation, the absorbance was measured at 275 nm. One fibrinolytic unit is defined as the amount of enzyme that induces an increase in absorbency at 275 nm equivalent to 1 μg of tyrosine per min at 37 °C.

### 2.7. Antioxidant Activity Assay

#### 2.7.1. Sample Extraction

Each sample (1 g) was extracted twice with 9 mL of 70% ethanol by shaking at 180 rpm for 3 h. After centrifuging the extract at 1628× *g* for 10 min, the supernatant was filtered through Whatman No. 2 filter paper and then freeze-dried.

#### 2.7.2. Total Phenolic Content (TPC)

TPC determination followed a method described by Singleton et al. [21], with modifications. One mL of each sample dissolved in the extracted solvent was mixed with 0.5 mL of 1 N Folin–Ciocalteu reagent and then 2.5 mL of 20% sodium carbonate. The mixture was incubated at room temperature for 40 min in the dark, and the absorbance was measured at 725 nm. The results are presented as gallic acid equivalent (GAE) in mg/g of sample.

#### 2.7.3. 2,2′-Azino-Bis (2-Ethyl Benzothiazoline-6-Sulphonate) (ABTS) Assay

The ABTS assay was conducted using the method described by Puchalska et al. [22], with modifications. An ABTS stock solution made from 7 mM ABTS and 2.45 mM potassium persulfate was incubated at 25 °C for 12–16 h in the dark. For analysis, the solution was diluted in methanol to obtain 0.70 ± 0.02 arbitrary units (AU) at 734 nm. Ten mg of sample was dissolved in 1 mL of extracted solvent. An amount of 10 μL of sample was mixed with 990 μL of diluted ABTS solution and then incubated at room temperature for 10 min in the dark. The absorbance was measured at 734 nm.

## 3. Results and Discussion

### 3.1. Comparison of MG (Mixed Grains) Metabolites during Fermentation

**Figure 1:**
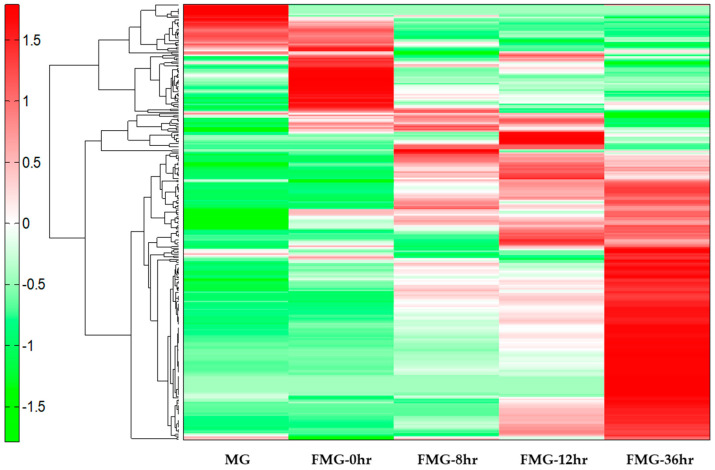
An hierarchical cluster analysis (HCA) heatmap comparing the metabolites from the mixed grains (MG) during fermentation. The x-axis shows MG (a grain mixture before steaming), fermented mixed grains (FMG)-0hr (a mixture of grain steamed for 30 min at 100 °C), FMG-8hr (a mixture of grain fermented for 8 h), FMG-12hr (a mixture of grain fermented for 12 h), and FMG-36hr (a mixture of grain fermented for 36 h) for each metabolite. The green and red colors indicate lower and higher contents of the metabolites, respectively.

The variation of the MG metabolites during fermentation was determined by CE-TOF-MS. Metabolomic analysis revealed a total of 276 peaks (165 metabolites in cation mode and 111 metabolites in anion mode) (File S1), which were annotated on the HMT’s standard library. To further classify related units in the analysis, a HCA with heatmap visualization was performed (Figure 1). The data showed that the growth of *Bacillus amyloliquefaciens* 245 in the presence of MG resulted in increased essential amino acids, bioactive polyamines (i.e., spermidine and agmatine), and phenolic compounds (i.e., homovanillic acid, 2.5-dihydroxybenzoic acid, and p-hydroxybenzoic acid).

**Figure 2:**
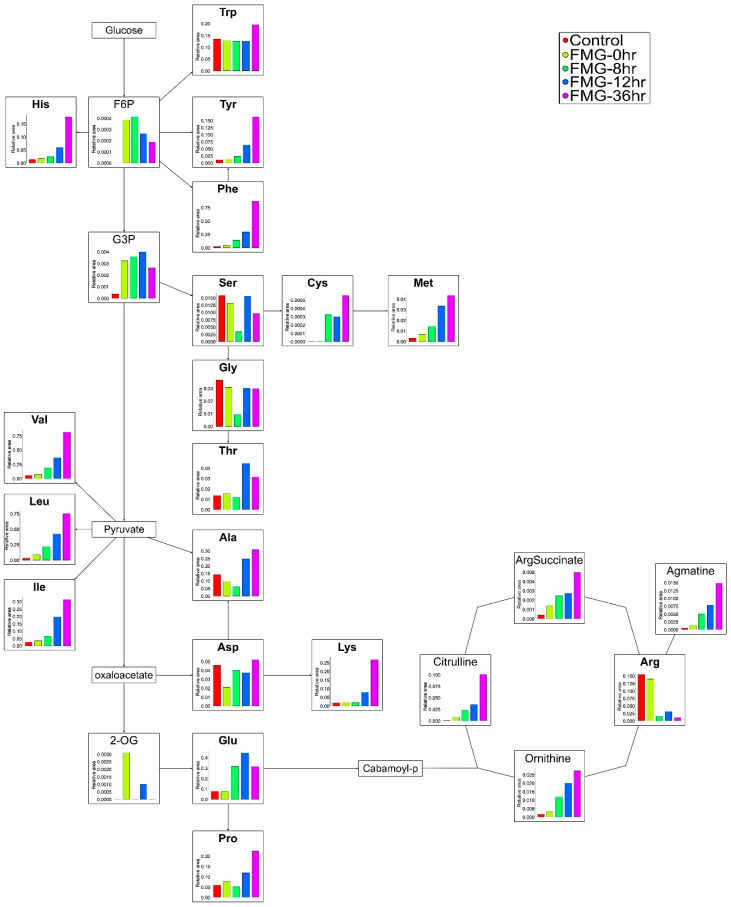
Scheme of the amino acid and the urea and polyamine pathways. The metabolic pathway was adopted from Kyoto Encyclopedia of Genes and Genomes (KEGG database. Changes in the levels of MG metabolites produced by *Bacillus amyloliquefaciens* 245 during fermentation were detected by capillary electrophoresis time-of-flight mass spectrometry (CE-TOF-MS). Each bar represents the relative metabolite abundance. Red, yellow, green, blue, and purple squares indicate MG (a grain mixture before steaming), FMG-0hr (a mixture of grain steamed for 30 min at 100 °C), FMG-8hr (a mixture of grain fermented for 8 h), FMG-12hr (a mixture of grain fermented for 12 h), and FMG-36hr (a mixture of grain fermented for 36 h), respectively.

The CE-TOF-MS-based metabolite profiling of the biological samples revealed noticeable changes in the components involved in amino acid pathways during fermentation (Figure 2). All the essential amino acids were increased with fermentation time. In non-essential amino acids, the contents of glutamic acid, cysteine, proline, and tyrosine were also gradually elevated during fermentation. Interestingly, the expression of alanine, aspartic acid, glycine, and serine was decreased at the middle phase of fermentation but elevated in the late phase.

The changes in the metabolites of the urea and polyamine pathway were shown to decrease the amount of arginine during fermentation (Figure 2). Meanwhile, the amount of citrulline, a precursor for the biosynthesis of arginine in the urea cycle, was increased. A similar ascending pattern was observed for the production of ornithine from arginine by arginase. The amount of agmatine transformed from arginine by arginine decarboxylase was also augmented with increasing fermentation time.

### 3.2. The Contents of Free Amino Acids during the Fermentation of MG

To validate the data for the amino acid metabolites during MG fermentation (File S1 and Figure 2), we quantitatively analyzed 18 amino acids at different fermentation stages.

Specifically, the total contents of amino acids were 368.76 mg/100 g in MG, 287.35 mg/100 g in FMG-0hr, 457.59 mg/100 g in FMG-8hr, 925.26 mg/100 g in FMG-12hr, and 1610.19 mg/100 g in FMG-36hr (Table 1). The contents of the essential amino acids such as histidine, isoleucine, leucine, lysine, methionine, phenylalanine, threonine, valine, and tryptophan in FMG-36hr increased about 4.5-, 9.0-, 7.1-, 9.5-, 1.5-, 18.9-, 2.2-, 12.8-, and 3.2-fold, respectively, compared to in MG. In the non-essential amino acids alanine, aspartic acid, glutamic acid, proline, serine, and tyrosine in FMG-36hr were 0.5-, 3.1-, 4.7-, 9.6-, 1.8-, and 10.1-fold, respectively, higher in quantity than in MG. However, the contents of arginine decreased from 76.9 mg/g in MG to 35 mg/g in FMG-36hr. The amount of glycine also decreased from 23.6 mg/g in MG to 14.3 mg/g in FMG-36hr. Additionally, no cysteine was detected during fermentation.

Consistent with these results, the fermentation of maize and other grains improved protein quality and increased the contents of lysine, methionine, and tryptophan [23]. Kang et al. [24] also found that fermented soybeans contained 11- and 28-fold greater contents of peptides and amino acid groups, respectively, than unfermented soybeans. In particular, the health benefits of the essential amino acids produced during fermentation have been extensively investigated. For example, phenylalanine, a precursor to tyrosine, is used for the biosynthesis of catecholamines that are known to be effective in depression, pain, and skin disorders [25]. Jansen et al. [26] also demonstrated that lysine was required for healthy tissue function, growth, calcium uptake, and healing as well as the immune system. In addition, leucine, isoleucine, and valine are branched-chain amino acids (BCAAs) that exhibit effective anti-fatigue and ergogenic activities in an animal model of power exercise training [27].

The contents of each essential amino acid continued to increase, showing a similar pattern to the data for the metabolites during fermentation. FMG with larger amounts of essential amino acids could be dietary supplements with health benefits, especially in the areas of muscle growth and the brain.

### 3.3. Content of Arginine Metabolites during Fermentation

**Figure 3:**
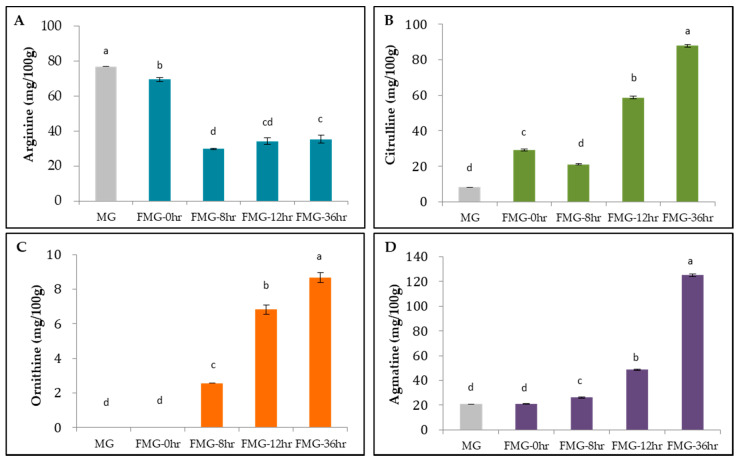
Arginine metabolites during fermentation. Content changes of (**A**) arginine, (**B**) citrulline, (**C**) ornithine, and (**D**) agmatine were measured using HPLC. MG, a grain mixture before steaming; FMG-0hr, a mixture of grain steamed for 30 min at 100 °C; FMG-8hr, a mixture of grain fermented for 8 h; FMG-12hr, a mixture of grain fermented for 12 h; and FMG-36hr, a mixture of grain fermented for 36 h. Data from one representative experiment of three independent experiments were presented as the mean with standard deviation of three technical replicates. Values with different letters are significantly different with *p* < 0.05 according to Duncan’s multiple-comparison test.

To confirm the differential patterns of metabolites in the urea cycle and polyamine pathways, we quantitatively analyzed arginine and its metabolites such as citrulline, ornithine, and agmatine during fermentation (Figure 3). As expected, the arginine contents of MG, FMG-0hr, FMG-8hr, FMG-12hr, and FMG-36hr gradually decreased to 76.9, 69.5, 29.8, 34.2, and 35.3 mg/100 g, respectively. The citrulline contents were 8.3 mg/100 g in MG, 29.1 mg/100 g in FMG-0hr, 21.1 mg/100 g in FMG-8hr, 58.8 mg/100 g in FMG-12hr, and 87.8 mg/100 g in FMG-36hr. The ornithine contents were 2.6 mg/100 g in FMG-8hr, 6.8 mg/100 g in FMG-12hr, and 8.7 mg/100 g in FMG-36hr, but no ornithine was detected in MG and FMG-0hr. The agmatine contents of MG, FMG-0hr, FMG-8hr, FMG-12hr, and FMG-36hr were 20.7, 20.8, 26.2, 48.6, and 125.0 mg/100 g, respectively.

The data imply that *Bacillus amyloliquefaciens* 245 modulated the arginine–deiminase system for arginine decomposition. Our speculation was supported by other investigators demonstrating that *Bacillus amyloliquefaciens* utilized arginine deiminase to effectively produce citrulline from arginine in fermented grains [28]. In addition to *Bacillus amyloliquefaciens*, *Lactobacillus brevis* and *Weissella confusa* showed arginine catabolism as well as citrulline and ornithine anabolism [29]. A comparable situation was also found in *Aspergillus oryzae*, widely used in the production of various Asian fermented foods [30].

There is growing evidence for the superior biological health benefits of arginine metabolites over arginine itself. A dietary supplement of citrulline malate increased endothelial-dependent vasodilation in healthy, normotensive, and physically active young adults [31]. Furthermore, L-citrulline supplementation increased nitric oxide (NO) availability, resulting in better protection of the aorta from senescence in diabetic rats than L-arginine supplementation [32]. Citrulline supplementation appeared to more efficiently increase arginine bioavailability than the supplementation of arginine itself in an animal study [33]. Ornithine, another arginine metabolite with therapeutic potential, has been demonstrated to modulate lipid metabolism and the urea cycle, leading to less fatigue in healthy subjects under physical load [34]. Agmatine has also been reported to improve the cardiovascular system by suppressing the inducible nitric oxide synthase (NOS)-mediated induction of NO synthesis [35].

*Bacillus amyloliquefaciens* 245 may therefore be a suitable microorganism for transforming arginine to citrulline, ornithine, and agmatine. The nutritional value of FMG consisting of a large number of arginine metabolites could make them attractive functional ingredients for maintaining cardiovascular health in a broad manner.

### 3.4. Enzymatic Activities in FMG

Amylase, protease, and fibrinolytic enzymes are traditionally produced by certain microorganisms in the fermentation process [2,10,36,37,38,39]. We examined whether the activity of amylase, protease, and fibrinolysis was affected by *Bacillus amyloliquefaciens* 245 in MG during fermentation.

**Figure 4:**
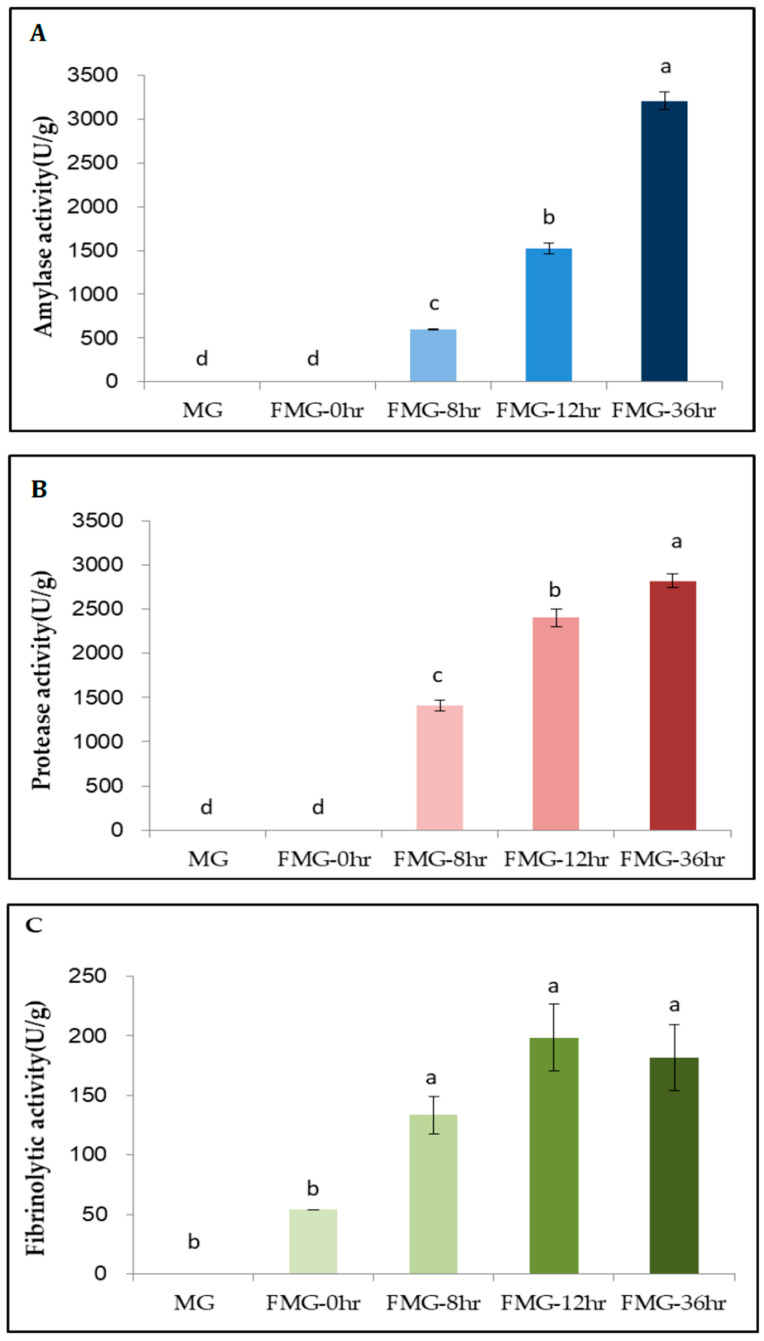
Enzymatic activity during MG fermentation. (**A**) Amylase, (**B**) protease, and (**C**) fibrinolytic activities were measured in each extracted sample. MG, a grain mixture before steaming; FMG-0hr, a mixture of grain steamed for 30 min at 100 °C; FMG-8hr, a mixture of grain fermented for 8 h; FMG-12hr, a mixture of grain fermented for 12 h; and FMG-36hr, a mixture of grain fermented for 36 h. Data from one representative experiment of three independent experiments were presented as the mean with standard deviation of three technical replicates. Values with different letters are significantly different with *p* < 0.05 according to Duncan’s multiple-comparison test.

We found that amylase activity was 0, 0, 594.0, 1525.8, and 3210.0 U/g in MG, FMG-0hr, FMG-8hr, FMG-12hr, and FMG-36hr, respectively (Figure 4A). Kunamnei et al. [37] demonstrated that the SSF of wheat bran by a thermophilic fungus at 50 °C for 120 h with an initial moisture content of 90% resulted in the highest amylase activity, at 534 U/g. We observed the maximum level of amylase activity (3210.0 U/g) in our mixture inoculated with a 10% ratio of *Bacillus amyloliquefaciens* 245 at 37 °C and 95% humidity for 36 h. Thus, further investigation is needed to determine whether bacterial microbiota rather than fungal communities are cost-effective for elevating amylase activity during fermentation with the same substances.

Under the same fermentation conditions, the protease activity of MG, FMG-0hr, FMG-8hr, FMG-12hr, and FMG-36hr was 0, 0, 1409.2, 2401.1, and 2818.6.0 U/g, respectively (Figure 4B). We confirmed that enzymatic activity was maximized at FMG-36hr. These data suggest that high proteolysis by *Bacillus amyloliquefaciens* 245 led to increased and balanced amounts of essential amino acids (Table 1). Consistent with our findings, *Bacillus amyloliquefaciens* U304 produced a highly active protease, increasing the degree to which large-molecular-weight proteins broke down into small-molecular-weight peptides and amino acids in fermented soybean meal [2,10].

A fibrinolytic enzyme that converts plasminogen to plasmin directly prevents intravascular coagulation without the risk of hemorrhage [40]. According to our results, the fibrinolytic activities induced by *Bacillus amyloliquefaciens* 245 in MG, FMG-0hr, FMG-8hr, FMG-12hr, and FMG-36hr were 0, 53.9, 133.3, 198.6, and 181.6 FU/g, respectively (Figure 4C). FMG-36hr displayed about 3-fold higher fibrinolysis than FMG-0hr (*p* < 0.05). Yao et al. [41] also demonstrated that *Bacillus amyloliquefaciens* RSB34 produced the strongest activity among nine Bacillus strains used as starters for traditional Korean fermented soy foods such as doenjang. Various microorganisms besides *Bacillus amyloliquefaciens* and substances have been used to produce fibrinolytic properties. For example, the fibrinolytic activity of roasted soybean flour and mixed grains (barley and brown rice) fermented by *Bacillus* spp. was higher than that of fermented roasted soybean flour [38]. In particular, Vijayaraghavan et al. [39] demonstrated that the highest fibrinolysis by *Bacillus cereus* IND1 was in wheat bran compared to different agroresidues including banana peel, tapioca peel, rice bran, and green gram husk.

Our results showed that MG with wheat bran, wheat germ, oats, brown rice, barley, and lentils inoculated with *Bacillus amyloliquefaciens* 245 displayed effective enzymatic activities for amylase, protease, and fibrinolysis.

### 3.5. Total Phenolic Content (TPC) and ABTS+ Radical Scavenging Capacity in MG during Fermentation

It is extensively established that the increased contents of polyphenols following grain fermentation are strongly correlated with their antioxidant activity [42]. We also measured the TPC and antioxidant activities in the MG during fermentation.

**Figure 5:**
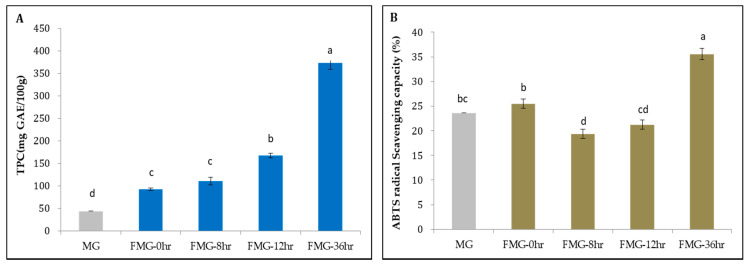
The contents and activities of antioxidant during MG fermentation. (**A**) Total Phenolic Content (TPC) and (**B**) ABTS+ radical scavenging capacity during fermentation. MG, a grain mixture before steaming; FMG-0hr, a mixture of grain steamed for 30 min at 100 °C; FMG-8hr, a mixture of grain fermented for 8 h; FMG-12hr, a mixture of grain fermented for 12 h; and FMG-36hr, a mixture of grain fermented for 36 h. Data from one representative experiment of three independent experiments were presented as the mean with standard deviation of three technical replicates. Values with different letters are significantly different with *p* < 0.05 according to Duncan’s multiple-comparison test. GAE: gallic acid equivalent.

As shown in Figure 5A, the TPCs in MG, FMG-0hr, FMG-8hr, FMG-12hr, and FMG-36hr were found to be 44.2, 92.5, 110.7, 167.5, and 373.5 mg GAE/100 g, respectively. FMG-36hr displayed about 8.4-fold higher TPC than MG, a statistically significant difference. The increase in TPC with the time of fermentation is due to the elevation of phenolic compounds including homovanillic acid, 2.5-dihydroxybenzoic acid, and α-hydroxybenzoic acid, which we observed by metabolomic analysis using CE-TOF-MS (File S1). These data indicate that during fermentation, bacterial enzymes are able to modify the grain constituents affecting the TPC.

In parallel with the TPC, the antioxidant activity during fermentation was measured via the ABTS+ assay. The ABTS+ radical scavenging capacity of MG, FMG-0hr, FMG-8hr, FMG-12hr, and FMG-36hr was found to be 23.6, 25.5, 19.4, 21.3, and 35.6%, respectively (Figure 5B). FMG-36hr had the highest radical scavenging activity, in association with the largest TPC. However, unlike for the TPC, we did not observe an incremental increase in ABTS+ activity with fermentation time. Cao et al. [43] demonstrated that while phenolic compounds with double hydroxyls on the flavonoid B-ring displayed strong antioxidant activity, those with a single hydroxyl group on the B-ring showed minimal free radical scavenging ability. This indicates that antioxidant activity can vary depending on the composition of the total phenol compounds. Furthermore, it has been reported that the essential amino acids of protein hydrolysates from Cucurbitaceae seeds can be a potent source of antioxidants [44]. Thus, the ABTS+ radical savaging capacity of MG and FMG may be determined by the number of amino acid metabolites in addition to the composition of phenolic compounds.

## 4. Conclusions

In this study, we established the conditions for the SSF of MG containing wheat germ, wheat bran, oats, brown rice, barley, quinoa, and lentils using our patented microorganism, Bacillus amyloliquefaciens 245. Under these conditions, we demonstrated that the number of essential amino acids and arginine metabolites incremented with fermentation time. Amylase, protease, and fibrinolysis were also gradually activated according to the fermentation stage. Furthermore, 36-hr cultured FMG exhibited significant increases in antioxidant capability and total polyphenol content compared to MG. Thus, we provide compelling evidence that the nutritional value and functional capacity of FMG produced by SSF in the presence of Bacillus amyloliquefaciens 245 could render them potential sources of dietary supplements.

## Figures and Tables

**Table 1:** The contents of free amino acids during the fermentation of MG.

Amino Acids	MG	FMG-0hr	FMG-8hr	FMG-12hr	FMG-36hr
	(mg/100 g)				
Essential Amino Acids					
Histidine	13.27 ± 0.31 ^c^	ND	13.88 ± 0.46 ^c^	26.47 ± 1.03 ^b^	60.37 ± 1.83 ^a^
Isoleucine	7.43 ± 0.21 ^d^	6.40 ± 0.72 ^d^	9.86 ± 1.51 ^c^	31.30 ± 1.01 ^b^	66.52 ± 1.8 ^a^
Leucine	19.74 ± 0.65 ^d^	14.17 ± 0.23 ^e^	33.44 ± 0.88 ^c^	70.39 ± 2.12 ^b^	139.21 ± 1.25 ^a^
Lysine	19.46 ± 1.05 ^d^	19.46 ± 0.11 ^d^	23.7 ± 0.89 ^c^	61.64 ± 1.74 ^b^	184.25 ± 1.12 ^a^
Methionine	16.19 ± 0.17 ^b^	ND	ND	13.45 ± 0.52 ^c^	24.21 ± 2.33 ^a^
Phenylalanine	12.65 ± 0.67 ^d^	9.34 ± 0.20 ^e^	39.86 ± 1.41 ^c^	81.46 ± 2.61 ^b^	239.46 ± 1.5 ^a^
Threonine	10.71 ± 0.73 ^b^	8.51 ± 0.15 ^b^	9.35 ± 1.65 ^b^	22.50 ± 1.18 ^a^	23.38 ± 2.25 ^a^
Tryptophan	13.92 ± 0.56 ^d^	11.40 ± 0.20 ^e^	34.61 ± 1.04 ^c^	78.95 ± 1.89 ^b^	178.23 ± 1.68 ^a^
Valine	29.00 ± 0.50 ^d^	32.00 ± 2.65 ^cd^	33.83 ± 0.76 ^c^	43.00 ± 2.00 ^b^	91.67 ± 1.53 ^a^
Non-Essential Amino Acids					
Alanine	36.49 ± 0.38 ^c^	26.47 ± 0.42 ^d^	23.36 ± 0.75 ^e^	91.32 ± 1.63 ^b^	113.88 ± 1.84 ^a^
Arginine	76.90 ± 1.30 ^a^	69.46 ± 0.29 ^b^	29.78 ± 1.88 ^d^	34.16 ± 2.32 ^c^	35.29 ± 1.69 ^c^
Aspartic acid	13.48 ± 0.80 ^d^	11.14 ± 0.09 ^e^	20.48 ± 1.04 ^c^	22.81 ± 1.30 ^b^	41.28 ± 2.13 ^a^
Glutamic acid	37.07 ± 1.37 ^d^	32.38 ± 0.77 ^e^	149.96 ± 1.89 ^c^	227.08 ± 2.52 ^a^	175.07 ± 0.9 ^b^
Glycine	23.61 ± 0.29 ^a^	15.29 ± 0.68 ^b^	6.78 ± 0.2 ^d^	15.26 ± 0.20 ^b^	14.30 ± 0.33 ^c^
Cysteine	ND	ND	ND	ND	ND
Proline	8.23 ± 0.21 ^e^	12.33 ± 1.53 ^d^	18.33 ± 1.53 ^c^	46.33 ± 3.21 ^b^	79.00 ± 1.00 ^a^
Serine	11.22 ± 0.86 ^b^	7.96 ± 0.55 ^c^	ND	10.73 ± 0.45 ^b^	20.21 ± 2.56 ^a^
Tyrosine	12.02 ± 0.53 ^d^	ND	18.56 ± 0.76 ^c^	44.69 ± 1.55 ^b^	121.38 ± 1.10 ^a^
Total	368.76	287.35	457.59	925.26	1615.07

Data from one representative experiment of three independent experiments were presented as the mean with standard deviation of three technical replicates. Different letters indicate significant differences at the *p* < 0.05 according to Duncan’s multiple-comparison test. MG, a grain mixture before steaming; FMG-0hr, a mixture of grain steamed for 30 min at 100 °C; FMG-8hr, a mixture of grain fermented for 8 h; FMG-12hr, a mixture of grain fermented for 12 h; and FMG-36hr, a mixture of grain fermented for 36 h. ND: not detected.

## Supplementary Materials

The following are available online at https://www.mdpi.com/2304-8158/9/11/1693/s1: File S1. Metabolic Profiling of FMG using *Bacillus amyloliquefaciens* 245.
